# Morse Code Recognition Based on a Flexible Tactile Sensor with Carbon Nanotube/Polyurethane Sponge Material by the Long Short-Term Memory Model

**DOI:** 10.3390/mi15070864

**Published:** 2024-06-30

**Authors:** Feilu Wang, Anyang Hu, Yang Song, Wangyong Zhang, Jinggen Zhu, Mengru Liu

**Affiliations:** 1 School of Electronic and Information Engineering, Anhui Jianzhu University, Hefei 230601, China; feiluw@mail.ustc.edu.cn (F.W.); hay0610@stu.ahjzu.edu.cn (A.H.); zwy1025@stu.ahjzu.edu.cn (W.Z.); zjg@stu.ahjzu.edu.cn (J.Z.); lmr@stu.ahjzu.edu.cn (M.L.); 2 Key Laboratory of Building Information Acquisition and Measurement Control Technology, Anhui Jianzhu University, Hefei 230601, China

**Keywords:** morse code, carbon nanotube (CNT), flexible tactile sensor, recognition, long short-term memory (LSTM), accuracy

## Abstract

Morse code recognition plays a very important role in the application of human–machine interaction. In this paper, based on the carbon nanotube (CNT) and polyurethane sponge (PUS) composite material, a flexible tactile CNT/PUS sensor with great piezoresistive characteristic is developed for detecting Morse code precisely. Thirty-six types of Morse code, including 26 letters (A–Z) and 10 numbers (0–9), are applied to the sensor. Each Morse code was repeated 60 times, and 2160 (36 × 60) groups of voltage time-sequential signals were collected to construct the dataset. Then, smoothing and normalization methods are used to preprocess and optimize the raw data. Based on that, the long short-term memory (LSTM) model with excellent feature extraction and self-adaptive ability is constructed to precisely recognize different types of Morse code detected by the sensor. The recognition accuracies of the 10-number Morse code, the 26-letter Morse code, and the whole 36-type Morse code are 99.17%, 95.37%, and 93.98%, respectively. Meanwhile, the Gated Recurrent Unit (GRU), Support Vector Machine (SVM), Multi-Layer Perceptron (MLP), and Random Forest (RF) models are built to distinguish the 36-type Morse code (letters of A–Z and numbers of 0–9) based on the same dataset and achieve the accuracies of 91.37%, 88.88%, 87.04%, and 90.97%, respectively, which are all lower than the accuracy of 93.98% based on the LSTM model. All the experimental results show that the CNT/PUS sensor can detect the Morse code’s tactile feature precisely, and the LSTM model has a very efficient property in recognizing Morse code detected by the CNT/PUS sensor.

## 1. Introduction

Flexible tactile sensors can achieve highly sensitive and precise recognition of tactile information, such as force, pressure, and shape changes, enabling machines to perceive and understand the external environment more intelligently [1]. In recent years, researchers have been attempting to use flexible sensors to acquire external information. They have designed flexible tactile sensors with different structures and materials to achieve high-sensitivity detection of various forces [2,3], enabling texture recognition [4,5] and temperature sensing [6]. Inspired by the fingerprints and epidermal structures of the skin, researchers have created stress sensors with porous structures or microstructures to further broaden the range of perceptual forces, increase sensitivity, and further enhance the performance of the sensors [7,8]. In addition, flexible sensors can sense the shape and type of the object, and real-time monitoring and recognition of the shape of an object can be realized by arranging flexible sensors on the surface of an object [9,10]. In the field of medical devices, sports monitoring, etc., human physiological signals can be monitored by flexible sensors [11,12,13]. With the rapid development of wearable device technology, flexible tactile sensors, as an important sensing technology, have attracted widespread attention in the field of wearable devices. Arranging tactile sensors on the hand or arm is used to validate the ability of tactile sensors to detect joint flexion, and in many works [14,15,16,17], the task of hand pose recognition is accomplished by supervised learning using tactile gloves. At the same time, tactile sensors can be used to sense hand-contact patterns, e.g., to categorize touch patterns [18], to recognize grasping objects [19], and to differentiate scratching behavior [20].

It is increasingly important to promote the application and development of flexible tactile sensor technology in the field of wearable devices. Song et al. [21] integrated six flexible pressure-sensor units into a wristband and achieved recognition of seven wrist gestures, five letter gestures, and eight sign language gestures with accuracies of 99.40%, 95%, and 98.44%, respectively. Gao et al. [22] integrated piezoresistive sensors and piezoelectric sensors into tactile gloves to realize the recognition of shape, size, and surface morphology of objects, and the recognition accuracy was 84%. Mekruksavanich et al. [23] used Inertial and Stretch sensors, combined with deep-learning techniques, to realize the recognition of human activity with a 97.68% recognition accuracy. Yu et al. [24] used MWCNTs/PDMS to achieve recognition of the four states (sitting, standing, walking, and running) with a recognition accuracy of 94%. For the research of flexible tactile sensor application processes, researchers try to use the flexible sensor on the recognition of Morse code. Adepu et al. [25] used two TeNWs/Ti_3_C_2_T_x_ nanohybrid-based sensors representing dot and dash signals, respectively, to achieve a wireless transmission of Morse code. Sadiq et al. [26] based their research on PDMS-CNTs film by detecting different human movements and recognizing Morse code and words.

In this paper, a flexible piezoresistive tactile sensor is designed and prepared based on the carbon nanotube (CNT) and polyurethane sponge (PUS) composite material to perceive 36 types of Morse code action precisely. To improve identification accuracy, the smoothing and normalization methods are applied to preprocess the raw time-sequential voltage signals collected from the CNT/PUS sensor for different Morse code. After that, the long short-term memory (LSTM) model with high feature-extraction property is constructed to recognize different Morse code signals, and excellent identification results are obtained. All the experiment results demonstrate that the CNT/PUS sensor can distinguish the Morse code feature signal precisely, and the LSTM model has a very efficient ability in recognizing the voltage time-sequential signals for the Morse code from the sensor.

## 2. Design and Preparation

### 2.1. Structure Design of the CNT/PUS Sensor

**Figure 1:**
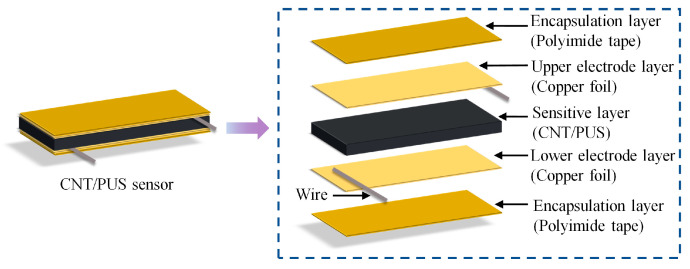
Schematic of the CNT/PUS sensor structure.

Based on the piezoresistive effect, a flexible tactile sensor consisting of five layers is designed, as shown in Figure 1. The sensitive layer is the core part of the tactile sensor, which is very sensitive to tactile pressure, and it is composed of the CNT/PUS composite with significant piezoresistive properties; the upper and lower electrode layers are made of copper foil with excellent conductivity and ductility; and the two encapsulation layers are made of polyimide tape with high-temperature resistance, corrosion resistance, and flexibility, which are used to protect the sensitive layer from being worn.

### 2.2. Preparation of the CNT/PUS Sensor

The key component of the flexible sensor is the pressure-sensitive layer, which is mainly composed of the CNT/PUS composite. In this paper, CNT material with excellent conductivity and stability is selected as the pressure-sensitive material of the flexible piezoresistive sensor. The performance parameters of the CNT solution used to make the CNT/PUS sensor are shown in Table 1.

The PUS is chosen as the flexible substrate material for the CNT/PUS composite in the sensitive layer, which is provided by Fushun Polyurethane Products Co., Ltd. (Yantai, China). The uniformly distributed porous structure of PUS makes it lightweight and highly elastic, making it the ideal material for CNT deposition.

**Figure 2:**
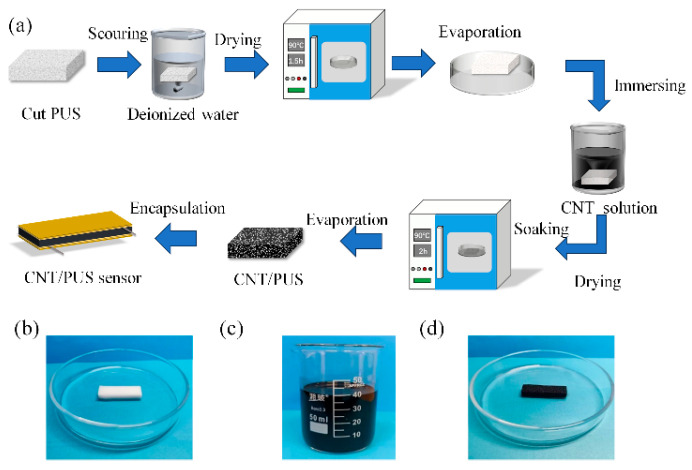
Preparation process of the CNT/PUS sensor. (**a**) The preparation of the CNT/PUS sensor: (**b**) polyurethane sponge; (**c**) carbon nanotube solution; (**d**) the carbon nanotube/polyurethane sponge.

The preparation process of the CNT/PUS sensor is shown in Figure 2. Firstly, the polyurethane sponge is cut into a size of 30 mm × 10 mm × 5 mm and washed with deionized water for 5 min to remove surface impurities, resulting in a clean polyurethane sponge. Then, the cleaned polyurethane sponge is placed in a drying oven set at 90 °C for 1.5 h to remove the residual moisture inside the sponge, resulting in a dried polyurethane sponge, as shown in Figure 2b. The dried polyurethane sponge is immersed in a carbon nanotube solution, as shown in Figure 2c, for 1 h to ensure uniform adsorption of carbon nanotube on the skeleton of the polyurethane sponge pores. After immersion, the carbon nanotube/polyurethane sponge is taken out and placed in a drying oven set at 90 °C for 2 h to remove the residual moisture in the sponge pore structure. Through the above steps, the carbon nanotube/polyurethane sponge shown in Figure 2d is obtained, which is used as the pressure-sensitive layer of the flexible tactile sensor. Attach copper foil to both ends of the carbon nanotube/polyurethane sponge and lead out the wires, fix them firmly with polyimide tape, and, finally, obtain the CNT/PUS sensor.

**Figure 3:**
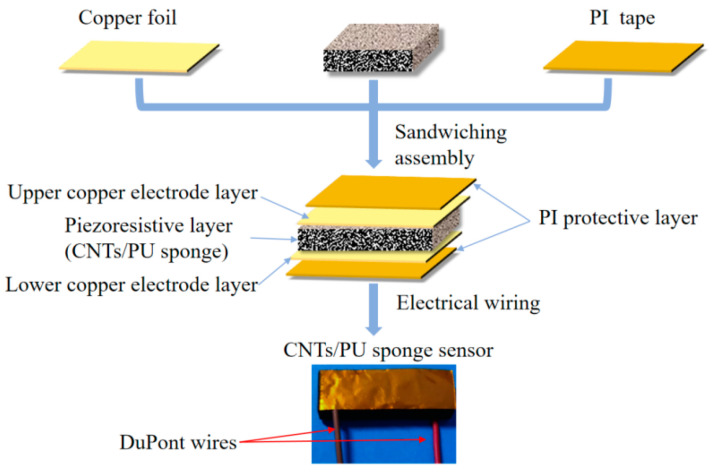
Encapsulation process of the CNT/PUS sensor.

Before the encapsulation process, the copper foil and polyimide tape were accurately cut to ensure their dimensions matched the carbon nanotube/polyurethane sponge’s size of 30 mm × 10 mm. Paste the cut copper foil and polyimide tape on the upper and lower surfaces of the CNT/PUS, and at the same time, lead the wires at the junction of the copper foil and the PU sponge, and press the whole sensor with a finger to make it stick firmly. The specific installation process is shown in Figure 3.

### 2.3. Performance Testing of the CNT/PUS Sensor

Based on our previous work [27], the CNT/PUS sensor had high sensitivity (2.7% kPa^−1^), prompt response (response/recovery time is 60/100 ms), and remarkable long-term stability (with the range of 0−100 kPa at 0.1 Hz for a period of 18,000 s). To verify the superior performance of the CNT/PUS sensor, a performance comparison of the sensor with the existing sensors in terms of material, preparation method, and sensor performance (sensitivity, response/recovery time, repeatability, and pressure range) has been done, as shown in Table 2. From the comparison in Table 2, it can be seen that the CNT/PUS sensor has better piezoresistive performance than the others.

To verify the deviation of the CNT/PUS sensor, deviation measurements were conducted on the prepared sensors. The specific equation is as follows:

(1) $$\delta_{L}=\frac{∆L_{max}}{Y_{FS}}\times 100\%$$

where $\delta_{L}$ represents the value of deviation in the sensor response, $∆L_{max}$ is the maximum deviation of the sensor’s output response, and $Y_{FS}$ is the maximum range of the sensor’s output response.

**Figure 4:**
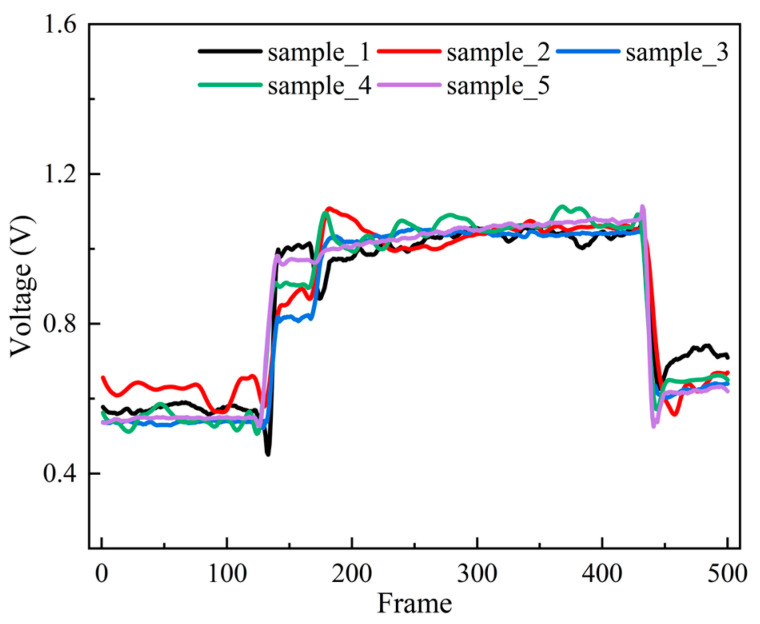
Response characteristic curve of the CNT/PUS sensor.

Five CNT/PUS sensors were obtained according to the same preparation process and encapsulation process. A pressure of 1 N was applied to each sensor for the same time and then withdrawn, and the voltage of the sensor outputs during this time period was recorded at all times. The voltage was recorded for the five sensor samples at 50, 150, 250, 350, and 450 frames, and the response curves are shown in Figure 4. Then, the sample standard deviation of the response time of the sample five sensors at 50, 150, 250, 350, and 450 frames was calculated, and the sample standard deviation was used to measure the degree of dispersion of each sample point with the following equation:

(2) $$S=\sqrt{\frac{1}{N-1}\sum_{I=1}^{N}{\left(X_{i}-\bar{X}\right)}^{2}}$$

where $\bar{X}$ is the sample mean, S is the sample standard deviation, and N is the number of samples.

According to Equation (2), the sample standard deviations for the five sensors at 50, 150, 250, 350, and 450 frames were calculated as 0.038 V, 0.075 V, 0.026 V, 0.011 V, and 0.0277 V, respectively. The maximum voltage output of the sensor was 1.06 V, the maximum deviation of the sensor’s output response $∆L_{max}$ was 0.075 V, and the maximum range of the sensor’s output response $Y_{FS}$ was 1.06 V. Therefore, according to Equation (1), the maximum deviation rate of the sensor samples $\delta_{L}$ was calculated to be 7.08%.

In summary, the CNT/PUS sensor prepared in this study exhibits excellent piezoresistive performance and low deviation, making it well-suited for the perception and detection of tactile signals. Based on the superior performance of this sensor, further research can be conducted on the high-precision detection of Morse code.

### 2.4. Sensing Mechanism of the Flexible Tactile Sensor

In this paper, a flexible tactile CNT/PUS sensor was prepared, and its performance has been analyzed. The aim of this paper is to investigate the sensing mechanism of the prepared CNT/PUS sensor.

**Figure 5:**
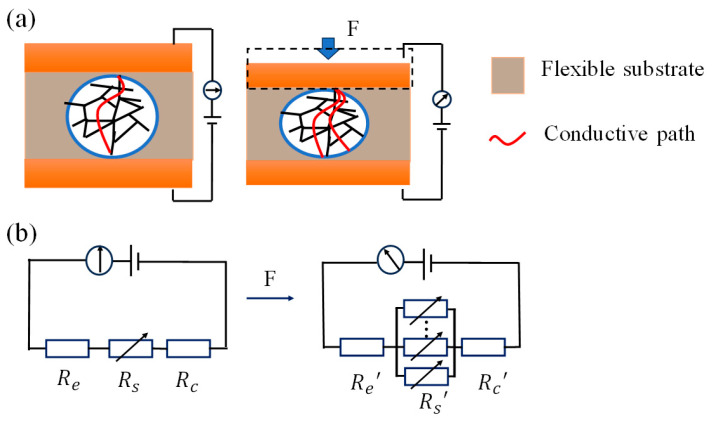
Mechanism of the CNT/PUS sensor: (**a**) microscopic mechanism; (**b**) macroscopic mechanism.

At the microscopic level, when an external force is applied to the CNT/PUS sensor, the structure of the sensor’s flexible substrate undergoes compression. This compression changes the number of conductive pathways in the internal structure, leading to a significant change in the sensor’s resistance, as shown in Figure 5a.

At the macroscopic level, assuming the total resistance of the CNT/PUS sensor is $R_{a}$. This total resistance is the sum of three components: the electrode layer resistance $R_{e}$, the piezoresistive layer resistance $R_{s}$, and the contact resistance $R_{C}$. Therefore, the total resistance of the sensor in its initial state can be represented by Equation (3):

(3) $$R_{a}=R_{e}+R_{s}+R_{C}$$

When the pressure was applied on the sensor, the electrode layer resistance *Re* remained almost unchanged due to the stability of its structure. However, the piezoresistive layer undergoes compression under pressure due to its porous structure, leading to a significant decrease in both the piezoresistive layer resistance *Rs* and the contact resistance *Rc*, as shown in Figure 5b. By analyzing the sensing mechanism of the CNT/PUS sensor, it is found that the change in sensor resistance is caused by the piezoresistive effect. This process converts mechanical pressure signals into electrical signals, which is of great significance for developing highly sensitive flexible tactile sensors.

The results indicate that the flexible tactile sensor, CNT/PUS sensor, prepared in this study is based on the piezoresistive effect. The performance of this sensor is closely related to its materials and structural design. These factors provide important bases for improving its performance and expanding the sensor’s application.

## 3. Data Acquisition for Morse Code Expression

This work aims to prepare a piezoresistive flexible tactile sensor for Morse code recognition. Tactile signals generated when expressing Morse code were captured using the CNT/PUS sensor, achieving accurate recognition of Morse code.

### 3.1. The Expression of Morse Code

**Figure 6:**
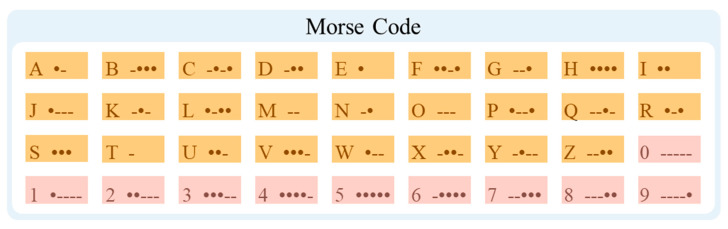
Morse code for letters of A–Z and numbers of 0–9.

In the Morse code system, a character is composed of short signals (dot signal “●”) and long signals (dash signal “-”), with different arrangements of signals representing different English letters, numbers, and punctuation marks. Specifically, the duration of a dot signal is one unit length, and the duration of a dash signal is three units in length. The interval between each signal is one unit in length, and the interval between each character is seven units in length. Different combinations of dot and dash signals form the basic symbol set of Morse code, as shown in Figure 6.

There are various forms of Morse code, including those based on light signals [32], electrical signals [33], and electrochromic signals [34], etc., and each form has its unique advantages and application scenarios as shown in Table 3. By comparing gesture coding with other forms of Morse code, the low cost and high flexibility of gesture coding have a unique advantage in the case of limited resources or restricted environments. In this paper, we use gesture coding to simulate Morse code signals.

This work proposes the use of index finger gestures to express the dot, dash, and interval signals of Morse code. Specifically, a dot signal is simulated by quickly tapping the index finger, a dash signal is simulated by sliding the index finger continuously in the air, and an interval signal is simulated by briefly pausing the index finger in the air. This gesture-based method of expression not only helps people communicate when electronic devices are unavailable but also serves as an effective method in sign language and other auxiliary communication techniques.

### 3.2. Data Collection for Morse Code from the CNT/PUS Sensor

During the data-collection process, experimenters performed gestures for 36 types (letters of A–Z and numbers of 0–9) of Morse code. Each Morse code gesture was repeated 20 times, and only 15 high-quality tactile data points were retained for each Morse code type. This strategy is used to prevent muscle fatigue and other incidental factors that could lead to erroneous data due to long-term repetitive operations by experimenters. By removing five low-quality Morse code tactile data points, a higher-quality Morse code dataset was created.

**Figure 7:**
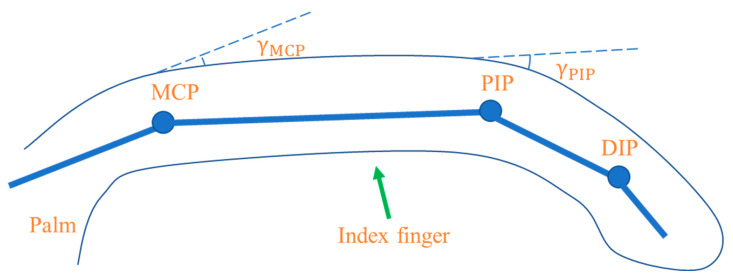
Illustration of the distribution of the joints in the index finger.

**Figure 8:**
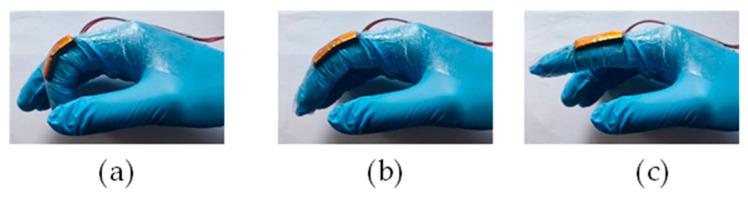
Simulating Morse code with the CNT/PUS sensor: (**a**) short taps motion; (**b**) long sliding motion; (**c**) short pause motion.

When simulating Morse code using gesture movements, the proximal interphalangeal (PIP) joint of the index finger (as shown in Figure 7) can effectively express Morse code information. During data collection, experimenters wear nitrile gloves, fix the CNT/PUS sensor at the PIP joint of the index finger, and perform the prescribed Morse code gestures, as shown in Figure 8. They also collect the timing voltage information output by the CNT/PUS sensor at the PIP joint of the index finger of nitrile gloves, and they simulate Morse code meanings through index finger gestures, as shown in Table 4.

The Arduino Mega 2560 board (Arduino Co., Ivrea, Italy) is used to collect the time-sequential voltage data output in real time from the CNT/PUS sensor for Morse code. Based on the dynamic response characteristics of the CNT/PUS sensor, the sensor sampling frequency is set to 180 Hz to capture rapidly changing tactile information. In this paper, we specify that each dot signal is expressed for 0.5 s, each dash signal is expressed for 1.5 s, and each interval signal is expressed for 0.5 s when expressing 36 types of Morse code characters using gesture action simulation. Specifically, the shortest expression time is for the letter E (“●”) at 0.5 s (including one dot signal), and the longest expression time is for the number 0 (“-----”) at 9.5 s (including five dash signals and four interval signals). To reduce errors caused by different expression times for individual letters or numbers and to improve Morse code encoding efficiency [35,36], the sampling time for each type of Morse code character (number/letter) tactile data sample is fixed at 10 s. The letter E (“●”) has an expression time of 0.5 s followed by a 9.5-s interval, and the number 0 (“-----”) has an expression time of 9.5 s followed by a 0.5-s interval. This configuration setting not only ensures sufficient tactile information is collected but also avoids data redundancy due to excessive time. Therefore, the collected time-sequential voltage data of Morse code is one-dimensional; it contains 1800 frames (or 1800 features).

To increase the diversity and robustness of Morse code tactile data samples, four experimenters (two males and two females, all with normal activity of the index finger joints) participated in the collection of Morse code tactile data. Before the experiment, all participants received standardized training to familiarize themselves with the operation methods of simulating Morse code expressions using the CNT/PUS sensor. The training content included an introduction to the basics of Morse code and gesture simulation experiments. During the experiment, each participant continuously completed the expression of 36 Morse code characters within the specified time, followed by a 30-s rest. This process was repeated 15 times until each of the four participants completed 15 rounds of high-quality data collection. The time-sequential voltage signals output by the sensor were recorded in real time. After completing the Morse code tactile data-collection experiment, a total of 2160 Morse code data samples were obtained (four experimenters × 15 valid repetitions for each Morse code × 36 Morse code).

## 4. Data Preprocessing

During the process of collecting Morse code data, there are various interfering factors. For instance, the CNT/PUS sensor may experience output fluctuations due to mechanical fatigue from prolonged operation. Additionally, issues such as data loss during transmission can also affect the integrity of the original time-sequential voltage information. Under the influence of these interference factors, the data may exhibit frequent and slight fluctuations and may show pulse signals in some data frames. To improve data quality and ensure that the collected data more accurately reflect the characteristics of Morse code, the original data will be subjected to data smoothing and normalization. This aims to reduce the impact of noise and outliers, making the Morse code tactile data more stable and reliable.

### 4.1. Data Smoothing for the Morse Code from the Sensor

The collected Morse code time-sequential voltage data are processed for smoothing, aiming to reduce the fluctuations caused by noise in the original sequential information. The Simple Moving Average (SMA) algorithm is used to preprocess and smooth the data in this work. The principle of the algorithm is to use the average of multiple samples within the window to represent the value of the current sample so as to reduce the impact of random noise on the signal. The specific equation is as follows:

(4) $$y\left(k\right)=\frac{x\left(k\right)+x\left(k-1\right)+x\left(k-2\right)+\cdots x(k-M+1)}{M}$$

where $y\left(k\right)$ represents the moving average value at time k, $x\left(k\right)$ represents the data point at time k, and M is the window size.

**Figure 9:**
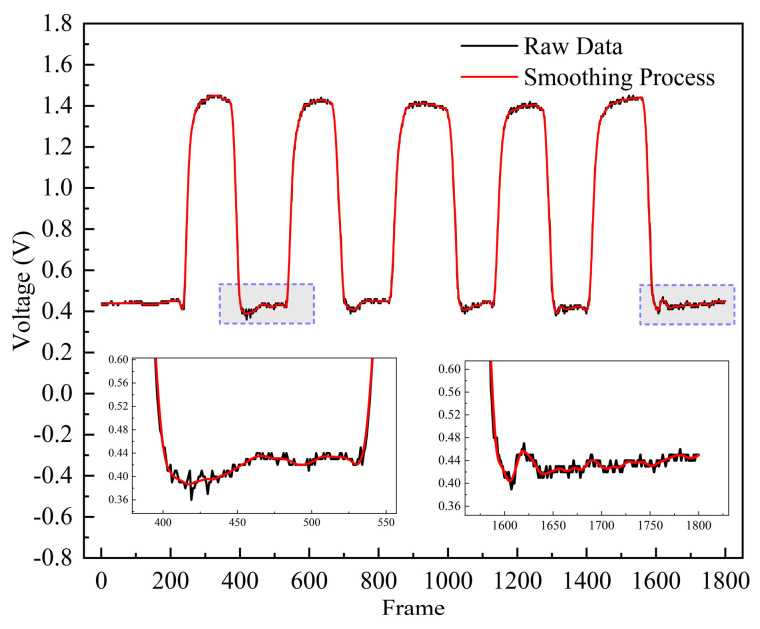
Raw data curve and data—smoothing curve.

The unprocessed raw data are compared to the smoothed data in Figure 9. The enlarged portion of the black curve in the figure reflects the excessive response of the sensor due to its own hysteresis effect. This response does not represent meaningful signals but rather errors caused by the sensor’s structure. The red curve in the figure presents the waveform of the data after smoothing, which reduces the noise level and smoothens the transitions in Morse code tactile data, enhancing the stability of the data. This preprocessing step lays a solid foundation for subsequent data analysis and Morse code recognition.

### 4.2. Normalization for the Morse Code from the Sensor

Normalization can prevent many problems such as slow training speed, gradient disappearance, or explosion in the training process of deep-learning models. Therefore, it is necessary to normalize the data after the data-smoothing process is completed. This Min–Max Normalization method is utilized to normalize the raw data in our work, which can reduce errors caused by differences in magnitude. The specific equation is as follows:

(5) $$X_{norm}=\frac{X-X_{min}}{X_{max}-X_{min}}$$

where $X_{norm}$ is the normalized value, X is the original data, $X_{min}$ is the minimum value in the original data, and $X_{max}$ is the maximum value in the original data.

**Figure 10:**
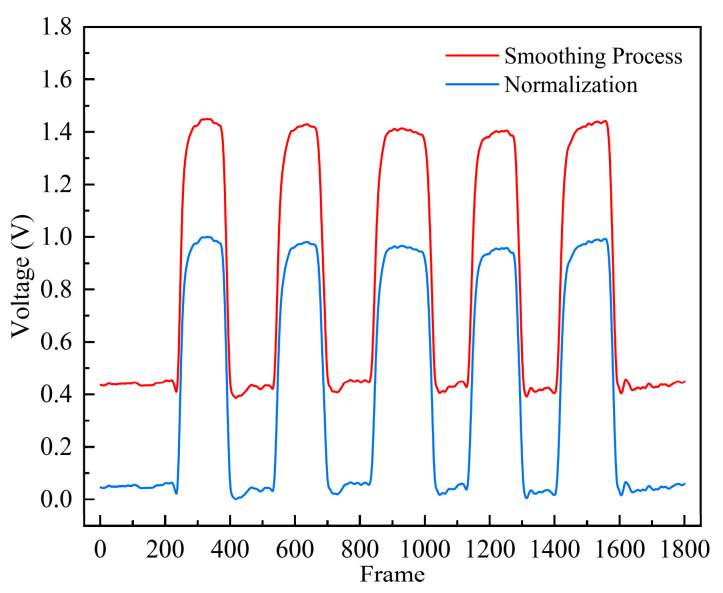
Data-smoothing curve and data-normalization curve.

As shown in Figure 10, the normalized data are mapped to the [0, 1] interval. This process not only enhances the data stability of the model training process but also helps accelerate the convergence speed of the model, effectively preventing overfitting during model training, thereby improving the performance of the model.

Through the completion of smoothing and normalization preprocessing on the tactile information of Morse code, the noise and outliers in the data have been effectively reduced, improving the data quality. As a result, the classification accuracy and processing efficiency of the Morse code recognition model are expected to be enhanced.

## 5. Morse Code Recognition Based on the LSTM Model

Through the tactile data-collection experiment of Morse code, we obtained the time-sequential voltage information under Morse code gestures. The Long Short-Term Memory (LSTM) model has shown excellent performance in handling sequence data with long-term dependencies. Considering the characteristics of the time-sequential voltage information of Morse code, this paper chooses to use the LSTM model for the recognition of Morse code.

### 5.1. Principle of the LSTM Network

**Figure 11:**
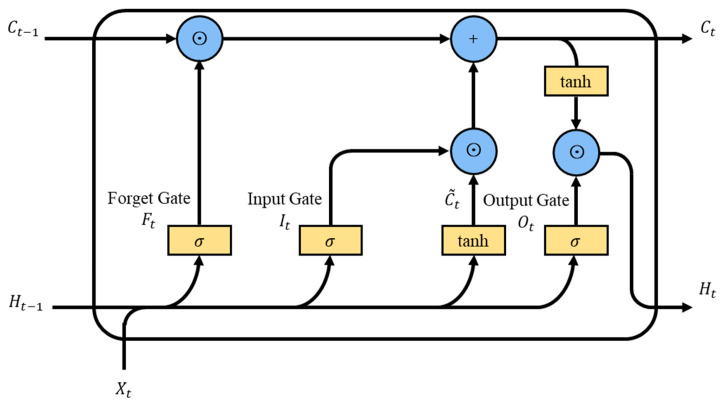
Schematic of the LSTM structure.

The LSTM network captures long-term dependencies in sequential data through the use of carefully designed internal states and gate mechanisms, enabling the effective learning of lengthy sequences [37]. At the core of the LSTM network is its unique memory unit, referred to as a cell, along with three crucial gate mechanisms: the forget gate, input gate, and output gate. These components work together to ensure the effective flow of time-sequential information and the retention of long-term memory, as illustrated in Figure 11. Within the LSTM unit, the information flow is primarily divided into two parts: one responsible for updating the cell state based on the control of the forget gate and input gate, and the other part performing autoregressive output computation under the control of the output gate.

The forget gate determines which information should be forgotten or discarded from the current cell state. Its calculation method, as shown in Equation (6), involves multiplying the input $X_{t}$ by its weight matrix $W_{xf}$, adding it to the previous time step’s hidden state $H_{t-1}$ multiplied by its weight matrix $W_{hf}$, then adding the bias vector $b_{f}$. Finally, the result is passed through the activation function $\sigma \left(x\right)$ as shown in Equation (7), yielding a value between 0 and 1. Here, the value 0 represents complete abandonment of the corresponding information in the old cell state, while the value 1 represents complete retention of this information.

(6) $$F_{t}=\sigma (X_{t}W_{xf}+H_{t-1}W_{hf}+b_{f})$$

(7) $$\sigma \left(x\right)=\frac{1}{1+\exp(-x)}$$

The input gate determines which new information will be stored in the cell state. Firstly, by multiplying the input $X_{t}$ by its weight matrix $W_{xi}$, adding it to the previous time step’s hidden state $H_{t-1}$ multiplied by its weight matrix $W_{hi}$, then adding the bias vector $b_{i}$, we obtain a value between 0 and 1 through the activation function $\sigma \left(x\right)$, as shown in Equation (8). At the same time, by multiplying the input $X_{t}$ by its weight matrix $W_{xc}$, adding it to the previous time step’s hidden state $H_{t-1}$, multiplied by its weight matrix $W_{hc}$, then adding the bias vector $b_{c}$, we obtain a new candidate memory cell ${\tilde{C}}_{t}$ through the activation function tanh(x), which represents the new information to be updated, as shown in Equation (9).

(8) $$I_{t}=\sigma (X_{t}W_{xi}+H_{t-1}W_{hi}+b_{i})$$

(9) $${\tilde{C}}_{t}=tanh(X_{t}W_{xc}+H_{t-1}W_{hc}+b_{c})$$

The output of the input gate $I_{t}$ is multiplied by ${\tilde{C}}_{t}$ to control how much of the new data from the candidate memory cell should be used. Then, the output of the forget gate $F_{t}$ is multiplied by $C_{t-1}$ to control the retention of information from the past memory cell. Finally, the two results are added together to obtain the updated cell state $C_{t}$ as shown in Equation (10). This design effectively alleviates the vanishing gradient problem and better captures the long-term dependencies present in sequences.

(10) $$C_{t}=F_{t}⊙C_{t-1}+{I_{t}⊙\tilde{C}}_{t}$$

The output gate controls how much information from the cell state $C_{t}$ will be used to generate the current hidden state $H_{t}$. Its calculation method is as follows: by multiplying the input $X_{t}$ by its weight matrix $W_{xo}$, adding it to the previous time step’s hidden state $H_{t-1}$ multiplied by its weight matrix $W_{ho}$, then adding the bias vector $b_{o}$, and, finally, passing the result through the activation function $\sigma \left(x\right)$ to obtain a value between 0 and 1, as shown in Equation (11). Next, this value is multiplied by the updated value of the cell state $tanh(C_{t})$ to obtain the final hidden state $H_{t}$, as shown in Equation (12).

(11) $$O_{t}=\sigma (X_{t}W_{xo}+H_{t-1}W_{ho}+b_{o})$$

(12) $$H_{t}=O_{t}⊙\tanh(C_{t})$$

Therefore, in performing the Morse code recognition task, the LSTM network, with its unique design structure, can effectively capture the long-term dependencies present in time-sequential tactile data, which is crucial for accurately recognizing the Morse code. This study chose the LSTM network model for Morse code recognition, aiming to fully leverage its advantages in handling time-sequential data, to achieve better classification accuracy and model generalization ability.

### 5.2. Construction of the LSTM Model for the Preprocessed Tactile Data

**Figure 12:**
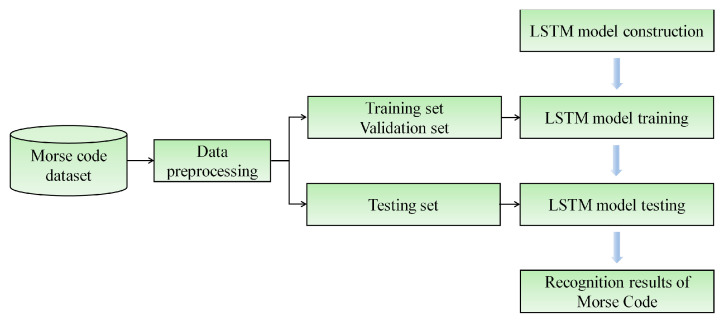
Flowchart of the training process based on the LSTM model.

In the paper, the time-sequential voltage information of simulated Morse code was collected using the CNT/PUS sensor, and the LSTM model was constructed to classify Morse code. The specific recognition process is shown in Figure 12.

**Figure 13:**
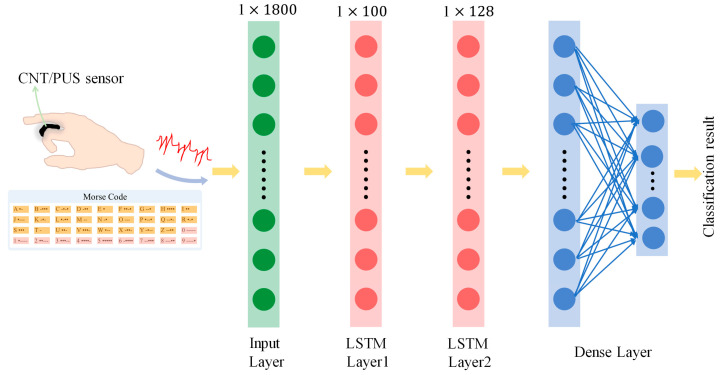
Flowchart of the LSTM model to recognize Morse code.

The LSTM model constructed in this paper is shown in Figure 13, which consists of two LSTM layers for feature extraction and a dense layer. The model takes input data from the tactile data-collection experiment of Morse code, including 36 types (letters of A–Z and numbers of 0–9) of Morse code, with each Morse code category providing 60 samples, totaling 2160 samples. Each tactile data sample is a piece of one-dimensional, time-sequential voltage information with 1800 features, which reflects the simulated Morse code information of the CNT/PUS sensor.

In the constructed LSTM model, LSTM network layers (each with 100 LSTM units) are used to extract and abstract deep features, which are then passed to the dense layer (with 36 neuron nodes) for further processing until the final Morse code classification task is completed. In this structure, the dense layer performs rich nonlinear transformations on the features extracted by the LSTM layer, enabling the model to capture more complex patterns in the tactile information. Through this multi-layered architecture, the LSTM model can effectively capture the long-term dependencies present in the tactile information of Morse code, providing an efficient and feasible solution for Morse code classification tasks.

### 5.3. Evaluation Metrics

In the multi-classification task of Morse code recognition, accuracy can only provide an overall overview of the classification performance cannot reflect specific information about false positives and false negatives, and it cannot provide detailed data on misclassifications. This work employs multiple evaluation metrics to comprehensively assess the performance of the LSTM model. Various evaluation metrics are used in this paper, including accuracy, precision, recall, and F1 score.

Accuracy refers to the proportion of correctly predicted samples to all samples by the model; precision refers to the proportion of samples predicted as the positive class by the model that are actually positive; recall refers to the proportion of actual positive samples that are correctly predicted as positive by the model; the F1 score is the mean of precision and recall, aiming to balance the relationship between these two metrics. The formulas for the selected evaluation metrics are as follows:

(13) $$Accuracy=\frac{TP+TN}{TP+TN+FP+FN}$$

(14) $$Precision=\frac{TP}{TP+FP}$$

(15) $$Recall=\frac{TP}{TP+FN}$$

(16) $$F1\text{-}score=2\times \frac{Precision\times Recall}{Precision+Recall}$$

where TP represents the number of samples that are truly positive and are predicted by the model as positive; FP represents the number of samples that are truly negative but are predicted by the model as positive; FN represents the number of samples that are truly positive but are predicted by the model as negative; TN represents the number of samples that are truly negative and are predicted by the model as negative.

### 5.4. Analysis of Recognition Results

#### 5.4.1. Recognition Results for Morse Code Based on the LSTM Model

The collected Morse code dataset is divided into 6:2:2 for training, validation, and testing sets. The LSTM model is used to perform Morse code classification on datasets with 10 types (Morse code numbers of 0–9), 26 types (Morse code letters of A–Z), and 36 types (Morse code numbers of 0–9 and letters of A–Z). For convenience, these three datasets are shortened as Morse_10, Morse_26, and Morse_36, respectively. Meanwhile, to illustrate the importance of the preprocessing process (smoothing and normalization), therefore, LSTM was used to recognize the preprocessed and unprocessed Morse code datasets (Morse_10, Morse_26, Morse_36), respectively, and the experimental results are shown in Table 5.

From the results in Table 5, comparing the results after data preprocessing and unprocessed data, it is found that the evaluation metrics of the results of unprocessed LSTM data for recognizing Morse_10, Morse_26, and Morse_36 are all lower than those after data preprocessing, thus indicating that the preprocessed data can improve the classification accuracy and generalization ability of the LSTM model, which proves the necessity of data preprocessing. From the results of the LSTM recognizing preprocessed data, it can be seen that the LSTM model achieved classification accuracies of 99.17%, 95.37%, and 93.98% on the testing sets of Morse_10, Morse_26, and Morse_36, respectively. Meanwhile, the LSTM model achieved a precision, recall, and F1 score of 94.32%, 93.98%, and 94.15% for Morse_36, which indicates that LSTM performs excellently in recognizing Morse code.

**Figure 14:**
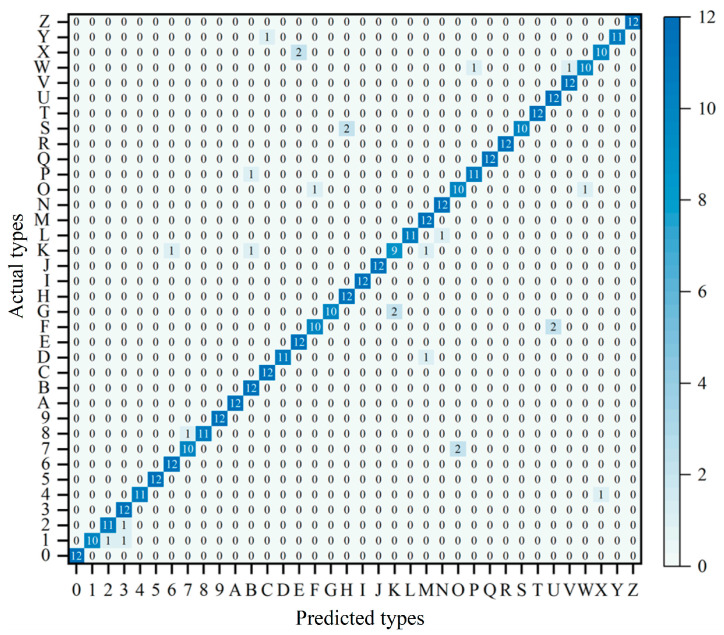
Confusion matrix for Morse code (36 types) recognition results.

To further analyze the performance of the LSTM model in Morse code recognition, the preprocessed Morse_36 is recognized using the LSTM model, the evaluation metrics of accuracy, precision, and recall are discussed in detail, and the classification results are as follows:

From the results in Figure 14, the overall accuracy of the LSTM model in recognizing Morse code (letters of A–Z and numbers of 0–9) is 93.98%. The accuracy of classification is generally higher than that of letter classification. This is because Morse code letters have higher morphological similarity, which increases the difficulty of differentiation and thus the difficulty of model classification.

**Figure 15:**
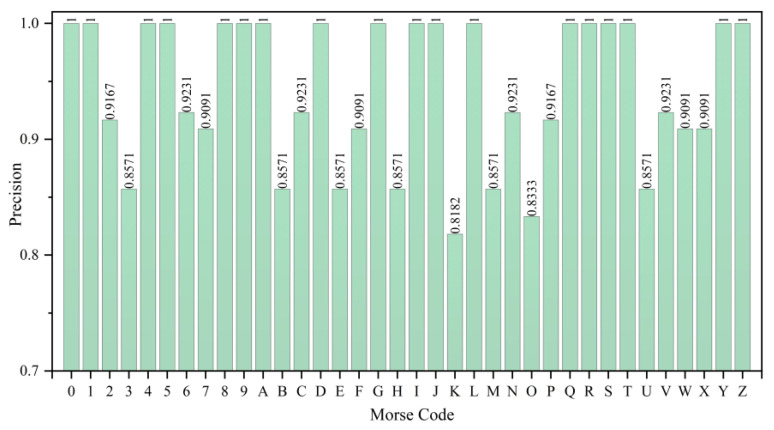
Precision of Morse code (36 types) recognition results.

From the precision results in Figure 15, the LSTM model achieves an average precision of over 85% for all 36 Morse code classes. Among them, 18 classes have a precision of 100%, but the precision for Morse code “K” and “O” is 81.82% and 83.33%, respectively. This indicates that the LSTM model has achieved excellent overall results in the Morse code recognition task (36 types), but there are certain shortcomings in the performance for certain types.

**Figure 16:**
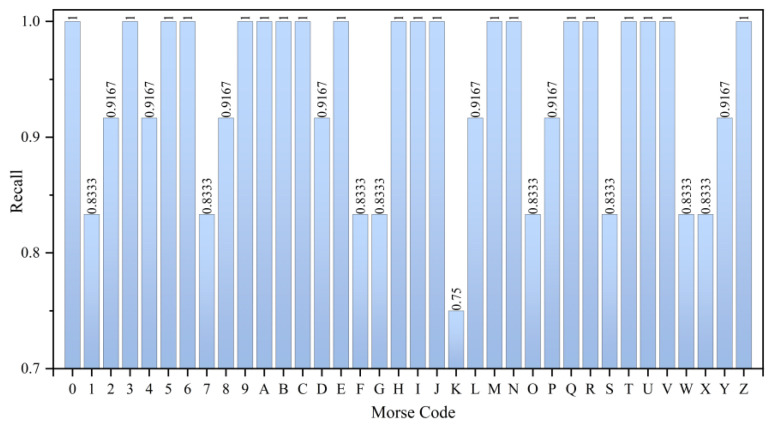
Recall of Morse code (36 types) recognition results.

From the results in Figure 16 regarding recall, the LSTM model achieves an average recall of 93.98% for the 36 Morse code classes. Specifically, the recall for 27 Morse code classes exceeds 90%, with 20 types having a recall of 100%. Compared to precision, more Morse code classes have lower recall values. This is because recall is less important than precision in the Morse code classification problem.

In summary, the LSTM model achieved an accuracy of 93.98%, a precision of 94.32%, a recall of 93.98%, and an F1 score of 94.15% in the classification of Morse code (36 types). Therefore, the LSTM model has achieved excellent classification results in the Morse code recognition task.

#### 5.4.2. Comparison Based on Different Models for Morse Code Recognition

**Figure 17:**
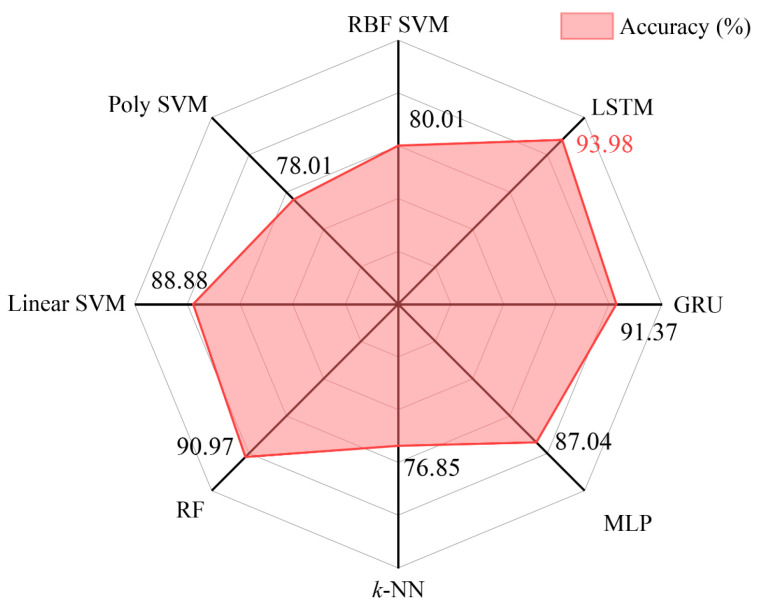
The accuracy of different models on the Morse code data-testing set.

Comparing the classification performance of different models on the same dataset can help reveal the strengths and limitations of each model, thereby providing a basis for selecting the most suitable model for a specific classification task. Based on the same preprocessed Morse code dataset (36 types), the classification performance of the Gated Recurrent Unit (GRU) model, Multi-Layer Perceptron (MLP) model, *k*-Nearest Neighbor (*k*-NN) model, Random Forest (RF) model, and Support Vector Machine (SVM) model were compared. The classification accuracy of each model on the testing set is shown in Figure 17.

From Figure 17, it can be seen that the LSTM model achieved the highest accuracy in Morse code classification, reaching 93.98% (as shown in the red text color in Figure 17), and the *k*-NN model has the lowest recognition accuracy at 76.85%. In our study, the data acquisition from the CNT/PUS sensor, although stable, may generate noise or errors under certain conditions, which may affect the recognition performance of some models. In particular, for models such as *k*-NN, which may rely more on the accuracy and consistency of the data, small fluctuations in the sensor may lead to a decrease in the recognition rate. The LSTM model has a stronger capability for handling time-series data, allowing it to better capture the sequential features in Morse code input. This enables LSTM to maintain a high recognition rate even in noisy conditions. Additionally, the LSTM model exhibits strong robustness to data fluctuations, effectively managing the uncertainties introduced by the sensor. A comparison was conducted between the CNT/PUS sensor and existing sensors in terms of model algorithms and accuracy. The results demonstrated that the sensor utilized in this study, when combined with the LSTM model, exhibited a high level of accuracy in recognizing Morse code, as shown in Table 6.

In order to better illustrate the effectiveness of using the LSTM model to recognize Morse code, the GRU, SVM, MLP, and RF models are established to recognize the three Morse code datasets Morse_10, Morse_26, and Morse_36 after data preprocessing, and the recognition results are shown in Table 7.

As shown in Table 7, the five different models have average accuracy rates for recognizing Morse code datasets (Morse_10, Morse_26, and Morse_36) in the following order: LSTM, GRU, RF, SVM, and MLP, with accuracies of 93.98%, 91.37%, 90.97%, 88.88%, and 87.04%, respectively. Comparing the evaluation metrics precision, recall, and F1 score, the scores from highest to lowest are LSTM, GRU, RF, SVM, and MLP. Through comparison, it is found that the LSTM model has the highest scores for the evaluation metrics, accuracy, precision, recall, and F1 score in recognizing the Morse_10, Morse_26, and Morse_36 datasets. From the Morse code dataset, including Morse_10, Morse_26, and Morse_36, it is found that all the models achieve the highest accuracy in recognizing Morse_10 and the lowest accuracy in recognizing Morse_36, and the models show consistency in recognizing the dataset. Through comparison, it is found that the LSTM model has better performance in recognizing Morse code classifications.

In summary, by comparing the models in Morse code recognition, the LSTM model shows higher recognition accuracy and stability, indicating its superior performance in Morse code recognition.

## 6. Conclusions

This study develops a flexible tactile sensor for recognizing Morse code. Initially, the CNT/PUS sensor is designed and prepared to express 36 types (letters of A–Z and numbers of 0–9) of Morse code gestures through different bending actions of the index finger. By collecting 2160 sets of Morse code gesture sequential voltage signals and combining them with the LSTM classification model, Morse code-recognition tasks are achieved. The results show that the LSTM model achieved a classification accuracy of 93.98% for 36 types of Morse code, with an average precision of 94.32%, an average recall of 93.98%, and an average F1 score of 94.15%. In conclusion, the LSTM model can effectively recognize tactile information outputs by the CNT/PUS sensor, enabling Morse code recognition. The innovation of this study lies in the material and structural design of the sensor, as well as the selection and application of the algorithm. By combining the CNT/PUS flexible tactile sensor with the LSTM algorithm model, the accuracy of Morse code recognition is not only improved but also demonstrates the potential of this method in the fields of flexible electronics and human–computer interaction.

## Figures and Tables

**Table 1:** The performance parameters of the CNT solution.

Properties	Value	Unit
quality score	2	%
purity	>98	%
length	10~30	μm
diameter	5~15	nm

**Table 2:** Performance comparison between the recently reported pressure sensors and this work.

Ref.	Materials	Preparation Method	Sensitivity (k·Pa^−1^)	Response/ Recovery Time (ms)	Repeatability (cycles)	Pressure Range (k·Pa)
This work	CNT/PUS	soaking-drying	0.027	60/100	18,000	0~100
[28]	MWCNT/PDMS	scraping-coating	0.026	320/170	2000	0~31.83
[29]	GNPs/MWCNTs/SR	dipping-coating	0.062	45/83	2000	0~4.5
[30]	MoS_2_/HEC/PUS	soaking-drying	0.746	120/120	2000	0~250
[31]	CuRGOMF	dipping-drying	0.088	300/NA *	5000	0~18

* Where “NA” is not mentioned in the paper.

**Table 3:** Advantages and disadvantages of different forms of Morse code.

Ref.	Principle	Advantages	Disadvantages
This work	Gesture-based	High flexibility	High complexity of recognition
[32]	Light-based	Long-distance transmission High-speed transmission	Environment-dependent High visualization requirements
[33]	Electrical	High stability Easy to implement	Limited distance Energy-consuming
[34]	Electrochromic	Low energy consumption Visually intuitive	Slow response speed Material limitations

**Table 4:** The meaning of Morse code simulated by index finger gestures.

Index Finger Behavior	Morse Code Meaning
Short taps motion	Dot signal
Long sliding motion	Dash signal
Short pause motion	Interval signal

**Table 5:** The recognition results for Morse code based on the LSTM model.

Dataset Status	Dataset	Accuracy	Precision	Recall	F1-Score
Unprocessed	Morse_10	90.74%	91.90%	90.74%	91.32%
	Morse_26	85.07%	86.12%	85.07%	85.59%
	Morse_36	82.18%	83.37%	82.18%	82.77%
Preprocessed	Morse_10	99.17%	99.70%	99.17%	99.43%
	Morse_26	95.37%	95.71%	95.37%	95.54%
	Morse_36	93.98%	94.32%	93.98%	94.15%

**Table 6:** Comparison of model algorithms and recognition accuracy between this work and others.

Ref.	Sensor	Model	Application	Accuracy
This work	CNT/PUS	LSTM	Morse code recognition	93.98%
[38]	Graphene/Nylon	Machine learning	Morse code recognition	>90%
[39]	Graphene aerogel	Machine learning	Gestures recognition	84.7%
[40]	CB/PDMS	LSTM + Dense	Gestures recognition	87.38%
[41]	CMOS sensor	YFDM	Morse code recognition	91%

**Table 7:** Recognizing Morse code with different models.

Model	Dataset	Accuracy	Precision	Recall	F1-Score
LSTM	Morse_10	99.17%	99.70%	99.17%	99.43%
	Morse_26	95.37%	95.71%	95.37%	95.54%
	Morse_36	93.98%	94.32%	93.98%	94.15%
GRU	Morse_10	98.33%	98.57%	98.33%	98.45%
	Morse_26	94.22%	94.61%	94.22%	94.41%
	Morse_36	91.37%	92.46%	91.37%	91.91%
SVM	Morse_10	94.17%	95.19%	94.17%	94.68%
	Morse_26	91.35%	92.29%	91.35%	91.82%
	Morse_36	88.88%	89.44%	88.88%	89.16%
MLP	Morse_10	92.50%	93.76%	92.50%	93.13%
	Morse_26	89.42%	90.42%	89.42%	89.92%
	Morse_36	87.04%	89.10%	87.04%	88.06%
RF	Morse_10	97.50%	98.00%	97.50%	97.75%
	Morse_26	94.87%	95.29%	94.87%	95.08%
	Morse_36	90.97%	91.68%	90.97%	91.32%

## Data Availability

The data used to support the study are available upon request to the corresponding author.
